# Genome-Wide Identification of Cassava Glyoxalase I Genes and the Potential Function of *MeGLYⅠ-13* in Iron Toxicity Tolerance

**DOI:** 10.3390/ijms23095212

**Published:** 2022-05-06

**Authors:** Fenlian Tang, Ruimei Li, Yangjiao Zhou, Shijia Wang, Qin Zhou, Zhongping Ding, Yuan Yao, Jiao Liu, Yajie Wang, Xinwen Hu, Jianchun Guo

**Affiliations:** 1 College of Tropical Crops, Hainan University, Haikou 570228, China; 19095131210051@hainanu.edu.cn (F.T.); liruimei@itbb.org.cn (R.L.); 18071010110007@hainanu.edu.cn (Y.Z.); 20086000210042@hainanu.edu.cn (S.W.); 21210710000025@hainanu.edu.cn (Q.Z.); 21220951310010@hainanu.edu.cn (Z.D.); wyj5501@hainanu.end.cn (Y.W.); 2 Institute of Tropical Bioscience and Biotechnology, Chinese Academy of Tropical Agricultural Sciences, Haikou 571101, China; yaoyuan@itbb.org.cn (Y.Y.); liujiao@itbb.org.cn (J.L.); 3 Hainan Institute for Tropical Agricultural Resource, Haikou 571101, China

**Keywords:** glyoxalase, iron toxicity, cassava, transgenic plants

## Abstract

Glyoxalase I (GLYI) is a key enzyme in the pathway of the glyoxalase system that degrades the toxic substance methylglyoxal, which plays a crucial part in plant growth, development, and stress response. A total of 19 *GLYI* genes were identified from the cassava genome, which distributed randomly on 11 chromosomes. These genes were named *MeGLYI-1–19* and were systematically characterized. Transcriptome data analysis showed that *MeGLYIs* gene expression is tissue-specific, and *MeGLYI-13* is the dominant gene expressed in young tissues, while *MeGLYI-19* is the dominant gene expressed in mature tissues and organs. qRT-PCR analysis showed that *MeGLYI-13* is upregulated under 2 h excess iron stress, but downregulated under 6, 12, and 20 h iron stress. Overexpression of *MeGLYI-13* enhanced the growth ability of transgenic yeast under iron stress. The root growth of transgenic *Arabidopsis* seedlings was less inhibited by iron toxicity than that of the wild type (WT). Potted transgenic *Arabidopsis* blossomed and podded under iron stress, but flowering of the WT was significantly delayed. The GLYI activity in transgenic *Arabidopsis* was improved under both non-iron stress and iron stress conditions compared to the WT. The SOD activity in transgenic plants was increased under iron stress, while the POD and CAT activity and MDA content were decreased compared to that in the WT. These results provide a basis for the selection of candidate genes for iron toxicity tolerance in cassava, and lay a theoretical foundation for further studies on the functions of these MeGLYI genes.

## 1. Introduction

Glyoxalase I (GLYI) is well-known for its functions as one of the main detoxifying agents of methylglyoxal (MG) produced by plants under stress [1]. Plant GLYIs are encoded by a multi-gene family, and the numbers of family members vary with species [1,2,3,4,5]. Four *VtGLYI* genes have been identified in the grape (*Vitis vinifera*) genome [2], 16 *BrGLYI* genes have been identified in the Chinese cabbage (*Brassica rapa*) genome [3], 24 *GmGLYI* genes have been identified in the soybean (*Glycine max*) genome [4], and 11 *OsGLYI* genes in rice (*Oryza sativa*) and 11 *AtGLYI* genes in *Arabidopsis thaliana* have been reported [5].

Plant GLYIs play essential roles in both biotic and abiotic stress tolerance [2,6,7,8,9]. In grapes, the *VvGLYI-1* gene is highly induced 48 h after downy mildew inoculation [2]. Overexpression of a glyoxalase-I gene (*PdGLX1*) from date palm (*Phoenix dactylifera*) in *Escherichia coli* can enhance its growth and MG detoxification ability. The *PdGLX1* gene can also supplement the functional deletion of the MG-hypersensitive glyoxalase 1 gene *–GLO1* (YML004C) yeast mutant, enhance the detoxification of MG, reduce the amassing of reactive oxygen species (ROS) under stress conditions, and promote yeast growth [9]. Several studies have reported the function of *GLYIs* in response and resistance to metal stress. In aluminum-treated tomatoes, a *GLYI* was recognized as a differentially expressed protein [10]. Overexpressing this gene can improve the tolerance of transgenic tomato plants to aluminum toxicity [7]. In Chinese cabbage, the expressions of *BrGLYI* genes in different heavy metal stresses have been analyzed, showing that the *BrGLYI1* gene was significantly induced by ZnD, *BrGLYI13* by ZnE, *BrGLYI15* by FeD, *BrGLYI8* by Cd, and *BrGLYI3*, *BrGLYI6*, and *BrGLYI11* by Pb [3]. In wheat, *TaGLYI* has been shown to be induced by ZnCl_2_, and its function in tolerance to ZnCl_2_ has also been verified by overexpression in tobacco [11].

Iron (Fe) is an important micro-metal element involved in various important processes of plant cells. However, excessive iron in the environment can hinder the growth and development of plants, affect the normal operation of carbon metabolism, enzyme activity, respiration, and photosynthesis, and produce ROS that cause oxidative toxicity [12,13]. The most obvious effects of iron toxicity on plant phenotypes are the inhibition of seed germination, the inhibition of the growth of roots and stems, and the reduction in yield. An amount of Fe higher than 4 mg/L reduces the germination of wheat seeds [14]. while the seed germination rate of weeds (*Echinochloa crusgalli*) is suppressed by 100 mg/L of Fe [15]. Apical roots are highly sensitive to iron and are the main area of iron toxicity, and excessive Fe attenuates root growth by increasing nitric oxide (NO) in the region of apical roots [16]. Nevertheless, when exposed to the same iron toxicity, tolerant rice cultivars can grow new lateral roots to rebuild their nutrient and water absorption systems, but sensitive varieties cannot [13]. Iron toxicity can reduce crop yields dramatically, such as that of rice, by up to 78% in West Africa [17]. Iron toxicity can also cause a reduction in crop spike number, spikelet sterility, and flowering delay or even failure [12,18]. In addition, under iron toxicity conditions, the activities of superoxide dismutase (SOD), peroxidase (POX), ascorbate peroxidase (APX), and glutathione reductase (GR) in the roots and shoots of iron-tolerant rice cultivars are significantly increased, while the activities of APX and catalase (CAT) in the roots of sensitive cultivars are significantly increased [19]. To regulate plant resistance to iron toxicity, some genetic data related to iron toxicity stress have been studied and obtained. Transcriptome analysis has revealed some genes related to iron homeostasis differentially expressed by iron toxicity in rice, such as *OsNAS3*, *OsVIT2*, *OsFer1*, and *OsFer2* [12,20]. HRZ ubiquitin ligase in rice is essential for protecting cells from the iron toxicity caused by excess iron [21].

Cassava (*Manihot esculenta*. Crantz) is a perennial woody shrub crop with starchy storage roots in the Euphorbiaceae family, planted in tropical and subtropical areas. Cassava is important both as food and for the production of bioenergy [22]. Cassava has a certain resistance to stress, such as tolerance to drought and barren soil [23,24]. More recently, cassava has been recognized for its role in rehabilitating land contaminated with heavy metals [25]. Cassava improves soil pH and absorbs excess soluble metals such as Cd, Cu, Pb, and Zn. More importantly, the heavy metals absorbed by cassava are mainly accumulated in leaves, fibrous roots, and stems, while the accumulation is the lowest in storage roots [25]. This feature makes cassava a suitable energy crop for the remediation of metal-contaminated land.

The intention of this study was to examine the hypothesis of whether the *GLYI* gene has a potential function in regulating the response of cassava to iron toxicity stress. First, we systematically identified and characterized cassava’s *MeGLYI* genes. Based on the expression patterns of said *MeGLYI* genes in cassava’s tissues/organs, *MeGLYI-13* was found to be mainly expressed in young tissues. Its expression profile in response to iron toxicity was then determined. The function of *MeGLYI-13* in excessive iron toxicity tolerance was further verified by transgenic yeast and *Arabidopsis*. This study is expected to provide important resources for further study of the functional characteristics of *MeGLYI* genes and their utilization for improving the stress response of this crop.

## 2. Results

### 2.1. Identification of 19 GLYIs in M. esculenta

In total, 19 GLYI proteins were identified based on BLASTP and PFMA searching, and the corresponding genes were designated as *MeGLYI-1–MeGLYI-19* based on their location on cassava’s chromosomes (Table S1). The physicochemical properties of these MeGLYIs were examined (Table S1). The CDS lengths of the *MeGLYIs* were between 354 bp (*MeGLYI-2*) and 1344 bp (*MeGLYI-3*). The protein lengths of the MeGLYIs varied from 117 aa (MeGLYI-2) to 447 aa (MeGLYI-3), the protein molecular weights distributed from 12.94 kDa (MeGLYI-2) to 49.29 (MeGLYI-3) kDa, and their isoelectric point (pI) ranged from 4.73 (MeGLYI-13) to 8.82 (MeGLYI-6).

**Figure 1:**
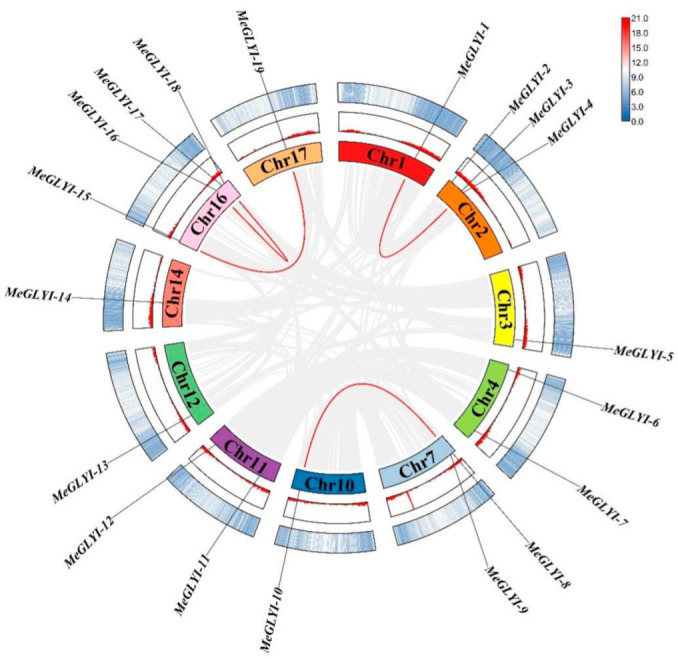
Distribution and collinearity analysis of the glyoxalase I gene family on chromosomes. Chr, chromosome; the gray line represents all of the collinear relationships in the cassava genome, while the red line represents the pairwise replication of the *MeGLYI* gene family.

The 19 *MeGLYI* genes were randomly located on 11 chromosomes of cassava (Figure 1). There was only one MeGLYI gene on Chr1, Chr3, Chr10, Chr12, Chr14, and Chr17; two on Chr4, Chr7, and Chr11; three on Chr2; and four on Chr16. The evolution process of the *MeGLYI* genes was explored through collinear analysis in the genome, and a total of four collinear relationships were found (Figure 1). The Ka/Ks value of the genes was calculated, which ranged from 0.09 to 0.79, meaning that negative selection was key to the evolution of the *MeGLYI* family (Table S2).

**Figure 2:**
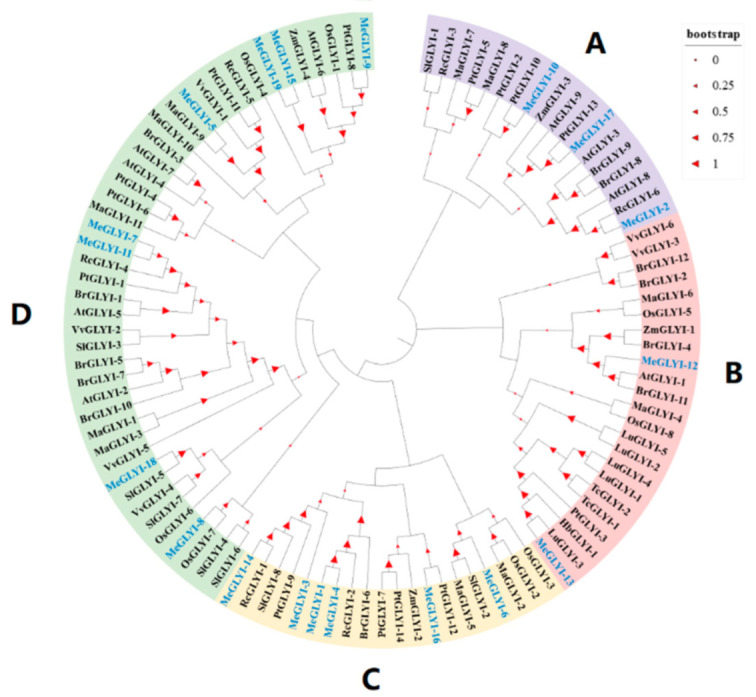
Phylogenetic tree of the GLYIs in cassava and other species. Me, *Manihot esculenta*; At, *Arabidopsis thaliana*; OS, *Oryza sativa*; Rc, *Ricinus communis*; Lu, *Linum usitatissimum*; Vv, *Vitis vinifera*; Br, *Brassica rapa*; Pt, *Populus trichocarpa*; Ma, *Musa acuminata*; Zm, *Zea mays*; Sl, *Solanum lycopersicum*; Tc, *Theobroma cacao*; and Hb, *Hevea brasiliensis*.

To learn the evolutionary relationships, the 19 MeGLYIs and GLYI protein sequences of 12 other plant species were aligned (Figure 2). The 19 MeGLYIs could be grouped into four subfamilies (A–D), of which subfamily D possessed the majority of the MeGLYI members (eight), followed subfamily C containing six members, subfamily A containing three members, and the subfamily B containing two members.

**Figure 3:**
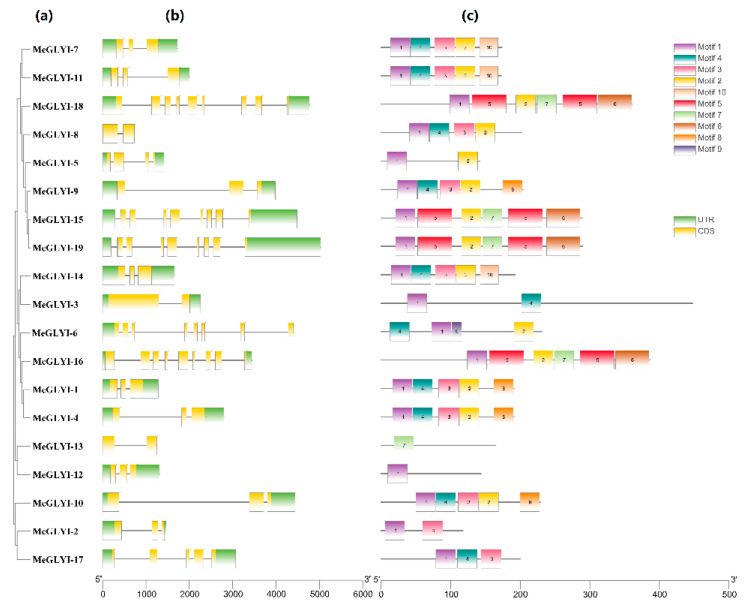
Analysis of gene structure and motifs according to the phylogenetic relationship. (**a**) Phylogenetic tree conducted based on the full-length sequences of cassava’s 19 MeGLYI proteins. (**b**) Exon–intron structure of cassava’s 19 MeGLYI genes. (**c**) The motif composition of cassava’s 19 MeGLYI proteins.

Gene structure analysis showed that the number of exons of the *MeGLYIs* ranged from two to nine, among which the maximum number of exons of *MeGLYI-15*, *MeGLYI-16*, *MeGLYI-18*, and *MeGLYI-19* was nine (Figure 3). The motif compositions of the 19 MeGLYI proteins were analyzed, and 10 distinct motifs were revealed (Figure 3). In accordance with the phylogenetic analysis, the closer the evolutionary relationship between the MeGLYIs, the more similar of the motif components.

### 2.2. Difference in the Expression of the MeGLYI Genes in Different Tissues/Organs of Cassava

**Figure 4:**
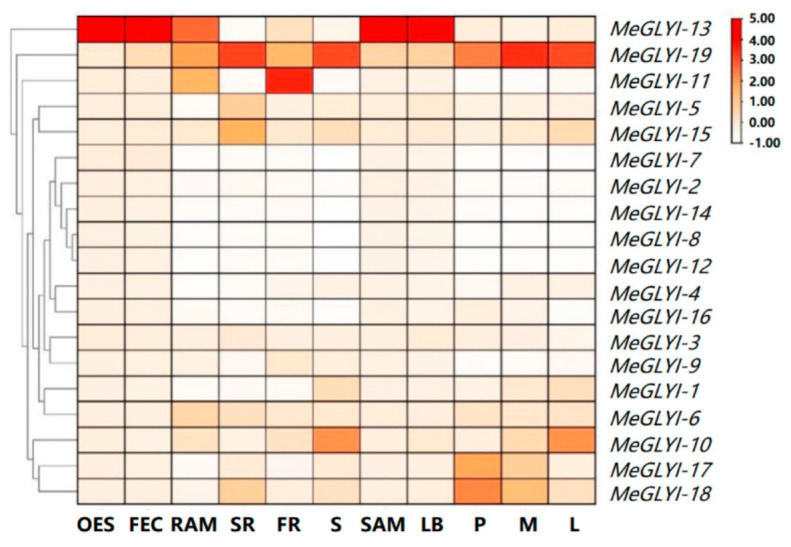
Expression analysis of the *MeGLYI* family genes in 11 tissues. OES, organized embryogenic structure; FEC, friable embryo callus; RAM, root apical meristem; SR, storage root; FR, fibrous root; S, stem; SAM, shoot apical meristem; LB, lateral bud; P, petiole; M, midvein; L, leaf.

To further detect the biological function of MeGLYIs, the expression patterns of the 19 *MeGLYI* genes were studied in the 11 tissues/organs of cassava, which were sourced from publicly available transcriptomic data. The *MeGLYI* genes were expressed in the 11 studied tissues/organs of cassava to varying degrees. In young tissues/organs such as organized embryogenic structures, friable embryo callus, root apical meristems, shoot apical meristems, and lateral buds, the expression level of *MeGLYI-13* was the highest. In the mature tissues/organs of storage roots, stems, petioles, midveins, and leaves, the expression level of *MeGLYI-19* was the highest. Meanwhile, in fibrous roots, the expression level of *MeGLYI-11* was the highest (Figure 4).

### 2.3. Expression of MeGLYI-13 in Response to Iron Stress

**Figure 5:**
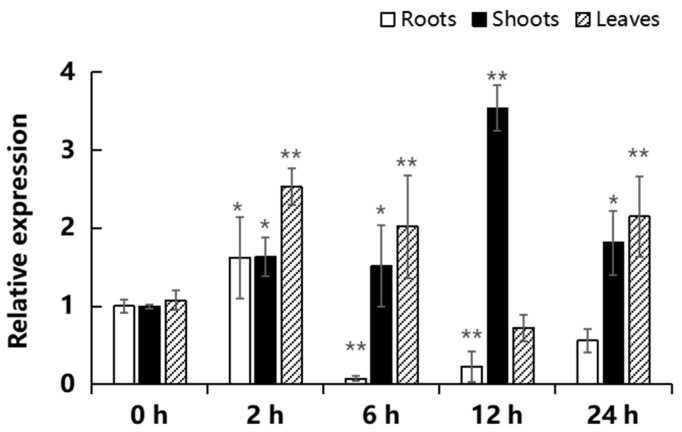
Expression profile of *MeGLYI-13* in the roots of cassava seedlings under iron stress. Values are means and standard deviations (n = 3). * indicates the significant difference *p* ≤ 0.05, ** indicates the significant difference *p* ≤ 0.01.

Since *MeGLYI-13* is preferentially expressed in young tissues and organs, we were curious to analyze whether it is involved in the stress response to iron. Cassava tissue culture seedlings at the one-month stage were subjected to treatment of 450 μmol/L of FeCl_3_ for 0, 2, 4, 6, 8, 12, and 24 h, separately. The mRNA levels of *MeGLYI-13* expressed in the roots, shoots, and leaves were analyzed by qRT-PCR. Under high-iron treatment, the expression of *MeGLYI-13* in the roots increased significantly at 2 h, but decreased significantly upon longer treatment (6, 12, and 24 h), and did not return to the normal level even after 24 h treatment (Figure 5). In the shoots, the expression of *MeGLYI-13* was significantly upregulated upon iron stress, in which the peak value was reached after 12 h of treatment. In the leaves, the expression peak value also appeared after 2 h of treatment (Figure 5).

### 2.4. Overexpression of MeGLYI-13 in Yeast Enhanced the Tolerance to Iron Stress

**Figure 6:**
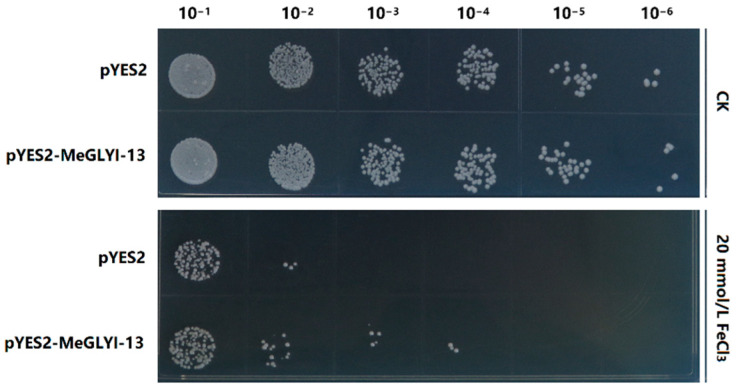
The functional validation of *MeGLYI-13* for iron stress resistance using transgenic yeast.

To investigate the function of *MeGLYI-13* in response to iron stress, the gene was expressed in INVSc1 yeast. Under normal conditions, no significant difference in growth ability was observed between the pYES2–*MeGLYI-13* and pYES2 transgenic yeasts. After treatment with 20 mmol/L of FeCl_3_, the pYES2–*MeGLYI-13* transgenic yeast grew better than the empty pYES2 vector transgenic yeast (Figure 6). These results indicate that overexpression of *MeGLYI-13* in yeast enhances the tolerance to iron stress.

### 2.5. Overexpression of MeGLYI-13 in Arabidopsis Raised the Tolerance to Iron Stress

**Figure 7:**
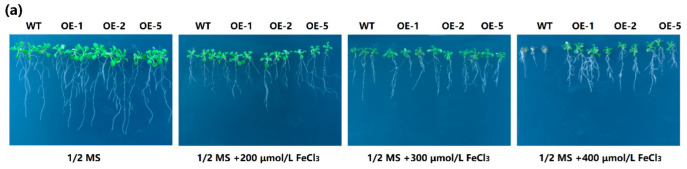

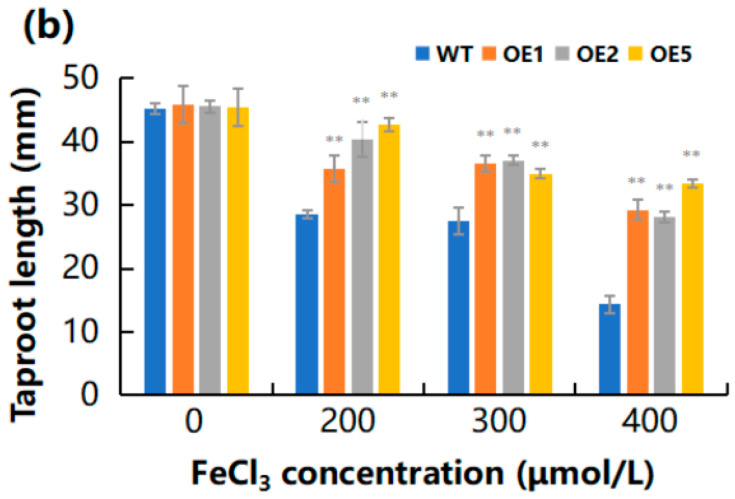
Functional validation of *MeGLYI-13* for iron stress resistance using the overexpression in *Arabidopsis*. (**a**) Phenotypes of the transgenic and wild-type *Arabidopsis* under different concentrations of FeCl_3_. (**b**) Taproot length statistics of the transgenic and wide-type *Arabidopsis* under different Fe concentration treatments. Values are means and standard deviations (n = 3). ** indicates the significant difference *p* ≤ 0.01.

To further verify the function of *MeGLYI-13* in coping with iron stress, the *MeGLYI-13*-overexpressing *Arabidopsis* was generated to study whether the expression of *MeGLYI-13* could enhance the iron tolerance (Figure 7). Three out of six independent transgenic lines (OE1, OE2, and OE5) were selected for iron treatment. Both the transgenic lines and the WT lines grew well on the ½ MS medium. The inhibition of a high level of FeCl_3_ (400 μmol/L) on the root growth of transgenic *Arabidopsis* seedlings was weaker than that of the wild type (Figure 7).

**Figure 8:**
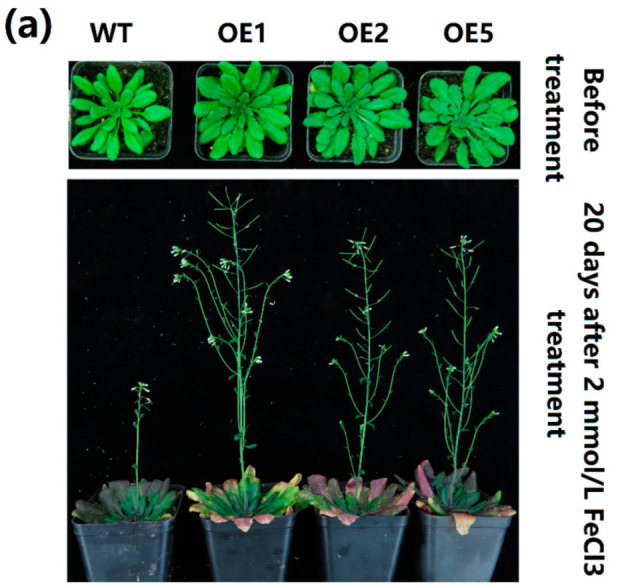

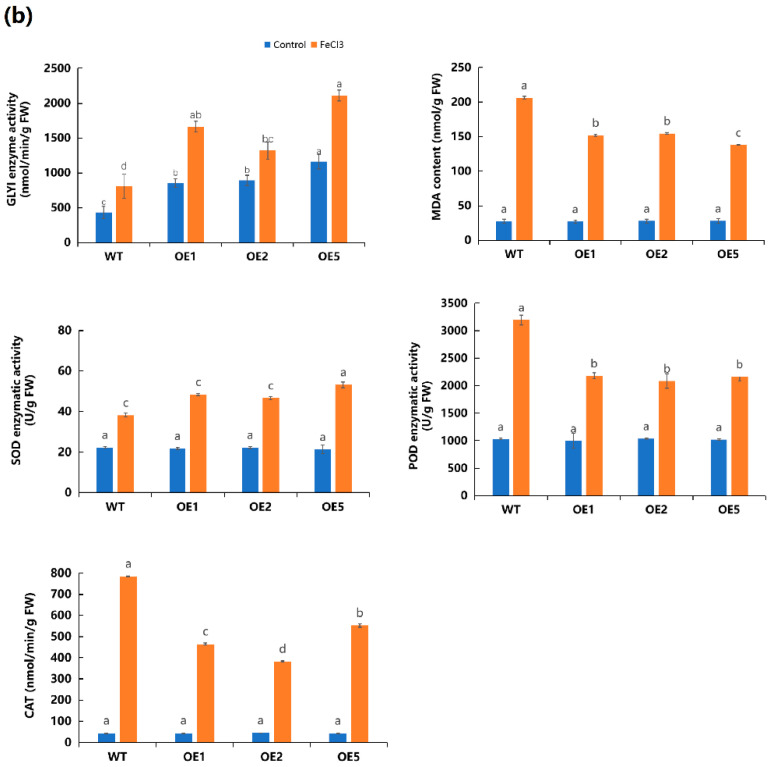
Tolerance of potted *MeGLYI-13* transgenic *Arabidopsis* to iron stress. (**a**) Comparison of phenotypic differences between the transgenic and WT *Arabidopsis* under 2 mmol/L of FeCl_3_ stress for 20 days. (**b**) The changes in GLYI, MDA, SOD, POD, and CAT in the transgenic and WT *Arabidopsis* under 2 mmol/L of FeCl_3_ stress for 20 days. Values are means and standard deviations (n = 3). The different letters (a, b, c and d) on the bars indicate the significant difference between the plant lines at *p* ≤ 0.05. WT, wild type.

To further understand the tolerance of transgenic *Arabidopsis* to iron stress, transgenic *Arabidopsis* and WT cultured in flowerpots were irrigated with 2 mmol/L of FeCl_3_ aqueous solution, and the growth changes were observed after 20 days of treatment (Figure 8). Under iron stress, the flowering and setting of the three transgenic *Arabidopsis* lines occurred earlier than those of the WT. The purple degree of old leaves in the transgenic plants was higher than that of the WT. The GLYI activity of the transgenic plants was significantly increased compared to that of the WT under non- or iron treatment conditions. The SOD activity of the transgenic *Arabidopsis* was significantly increased compared to that of the WT under iron stress conditions, while the MDA content and POD and CAT activity were lower than those of the WT, indicating that transgenic *Arabidopsis* suffered less stress damage. These results suggest that *MeGLYI-13* could indeed play a role in resistance to excessive iron stress.

## 3. Discussion

The GLYI family was recognized in cassava for the first time in this work, and the number (19) of gene members was more than that of grapes (4), *Arabidopsis* (11), rice (11), and Chinese cabbage (15) and less than that in soybean (24) and *Medicago truncatula* (29) [2,3,4,5,26]. Thus, species differences exist in terms of the size of the *GLYI* gene family. MeGLYI members can be divided into four subfamilies. The genes grouped into the same subfamily may have similar functions. In this study, the closer the MeGLYIs were to one another, the more similar their motif composition was, which further verified the reliability of the MeGLYI classification.

In previous studies, *GLYI* gene expression was found to be tissue- or developmental stage-specific [2,3,4,26]. In grapes, the expression level of *VvGLYI-1* in leaves, *VvGLYI-2* in leaves and tendrils, *VvGLYI-3* in tendrils, and *VvGLYI-4* in young ovules (20 days after flowering) was the highest [2]. In Chinese cabbage, the expression of *BrGLYI6* and *BrGLYI11* was higher in siliques, *BrGLYI9* was higher in silique walls, *BrGLYI5* and *BrGLYII2* were higher in roots, *BrGLYI8* and *BrGLYI11* were higher in flower buds, and *BrGLYI1*, *-7*, *-10*, *-14*, and *-15* were higher in callus [3]. In *M. truncatula,* the expression level of *MtGLYI-4* was the highest in all 17 tissues analyzed, while the expression level of *MtGLYI-3, MtGLYI-18*, and *MtGLYI-20* was low in all tissues. The expression of *MtGLYI-4*, *MtGLYI-12*, *MtGLYI-13*, *MtGLYI-14*, *MtGLYI-15*, *MtGLYI-23*, and *MtGLYI-24* was higher in young vegetative buds [26]. Previous studies have shown that GLYI activity is related to the cell division of plant callus [3]. In our work, the expression of cassava *MeGLYIs* also showed different tissue specificity. More interestingly, the expression level of *MeGLYI-13* was the highest in young tissues/organs such as organized embryogenic structure, friable embryo callus, root apical meristems, shoot apical meristems, and lateral buds. These results indicate that *MeGLYI-13* may participate in the process of cell division.

In recent years, enhanced GLYI activity has been associated with improved plant adaption to metal stress. The synergistic application of potassium and melatonin alleviates the toxic effect of Cd stress by increasing the activity of glyoxalase I in tomato seedlings [27]. In sugarcanes, a novel glyoxalase I gene (*SoGloI*) has been identified, which is upregulated by CuCl_2_, NaCl, CdCl_2_, and ZnSO_4_ [28]. The recombinant *Escherichia coli* expressing *SoGloI* has been shown to increase tolerance to high concentrations of CuCl_2_, CdCl_2_, NaCl, and ZnSO_4_ [28]. In wheat, *TaGLYI* has been isolated and shown to be induced by a high concentration of ZnCl_2_ [11]. After the overexpression of *TaGLYI* in tobacco leaves, the tolerance of transgenic tobacco to ZnCl_2_ stress has been shown to be significantly enhanced [11]. Meanwhile, NaCl and ZnCl_2_ stress induces *OsGLYI* expression in rice seedlings. The overexpression of the *OsGLYI* gene increases glyoxalase activity, decreases the MG level, and enhances the tolerance to NaCl and ZnCl_2_, while the seed setting rate and yield were shown to be higher than those of CK [29]. In another study, *SlGLYI*-overexpressing tomatoes showed significantly increased glyoxalase activity in their root tips under both Al and non-Al treatments, and the root fresh and dry weight were higher than those of the control [7]. In Chinese cabbage, the expression of *BrGLYI8* and *BrGLYI6* has been shown to be induced by Pb and Cd treatments [3]. However, the role of the *GLYI* gene in excessive iron stress has not been reported.

Previous studies have shown that apical roots are a major area of iron (Fe) toxicity and are highly sensitive to iron [16]. In this study, the *MeGLYI-13* gene from cassava was found to be the most highly expressed in the root tips of cassava. Therefore, *MeGLYI-13* was selected for further study in terms of the iron toxicity response. Consistent with our hypothesis, its expression was significantly induced by FeCl_3_ stress after 2 h of treatment. Its function in regulating iron stress tolerance was demonstrated by expression in yeast and *Arabidopsis*. Further experiments will be needed to reveal the molecular mechanism of *MeGLYI-13* in regulating the tolerance to iron toxicity. 

## 4. Materials and Methods

### 4.1. Identification and Analysis of GLYI Genes in Cassava

In the cassava genome database (Manihot esculenta V6.1), GLYI family genes in cassava were searched and identified in multiple ways, by searching the keywords glyoxalase, PF00903, PF12681 and PF18029, and BLAST based on the known GLYI protein sequences in *Arabidopsis thaliana*. The identified genes were named in ascending order according to their position on the chromosome. The physical and chemical properties of the genes were predicted using the ProParam online website. The chromosome position and collinearity of cassava’s MeGLYI genes were visualized by TBtools software v.1.089. The Ka/Ks values were calculated by KaKs_Calculator software v.2.0. The GLYI protein sequences in cassava, *Arabidopsis*, rice, castor bean (*Ricinus communis*), flax (*Linum usitatissimum*), grape (*Vitis vinifera*), Chinese cabbage, poplar (*Populus trichocarpa*), banana (*Musa acuminata*), corn (*Zea mays*), tomato (*Solanum lycopersicum*), cocoa (*Theobroma cacao*), and rubber tree (*Hevea brasiliensis*) were collected for phylogenetic analysis. MEGA 11.0 software was used to perform a multi-sequence comparison on the proteins. The neighbor-joining method was used to construct a phylogenetic tree. The test of phylogeny was used Bootstrap method with the number of bootstrap replications was 1000. The substitution model was Poisson model. The Gaps/Missing data treatment was Pairwise deletion. The GSDS website was used to illustrate a diagram of the structure of the *MeGLYI* genes. MEME was made to find and illustrate the conservative motifs. 

### 4.2. Expression Analysis of MeGLYIs in Different Tissues and Organs

Transcriptome data from 11 tissues/organs (fibrous roots, storage roots, shoots, leaves, petioles, medial veins, lateral buds, root apical meristems, shoot apical meristems, organized embryogenic structure, and friable embryo callus) of cassava were used in this study (Bioproject ID PRJNA324539) [24]. The FPKM values of all of the *MeGLYIs* were selected and converted into log2-fold, and then heat maps were draw by TBtools software [30].

### 4.3. Expression Analysis of MeGLYI-13 in Response to Iron Stress

To explore the function of the *MeGLYI-13* gene in iron stress, 40-day-old SC8 cassava seedlings were treated with 450 μmol/L of FeCl_3_ to induce excessive iron stress. Meanwhile, 40-day-old SC8 cassava seedlings without treatment were used as the control. Root, shoot, and leaf samples were harvested at 0, 2, 6, 12, and 24 h after the iron treatment, separately. In this experiment, two plants and three biological replicates were set for each treatment. Then, all of the samples were frozen by liquid nitrogen for isolating the total RNA using a Plant Total RNA Isolation Kit Plus (Foregene). The MonScript™ RTIII Super Mix with dsDNase (Two-Step) Kit was used to remove the remaining DNA from the RNA and then for reverse transcription of the RNA into cDNA. The qRT-PCR data were reacted by SYBR^®^ Premix Ex TaqTM II reagent (Takara) and detected by the ABI 7900 HT Fast Real-Time PCR System. The relative expression levels of *MeGLYI-13* at different times after treatment were calculated and analyzed by the popular 2^−ΔΔCT^ method. The primers used in this experiment are listed in Table S3.

### 4.4. Cloning of MeGLYI-13 and Functional Analysis in Yeast and Arabidopsis

The full-length sequence of *MeGLYI-13* was amplified from SC8 cassava using the primers *MeGLYI-13* F: GCTAGGGCTTCTCTGCTCTG and *MeGLYI-13* R: TCCCACAATCTGACACCTGC. PCR reaction was performed using Prime STAR H5 premix (Takara). The target fragment of the cloned *MeGLYI-13* gene was purified and recovered using a Gel Extraction Kit (Omega), and then sequenced.

A *KpnI* restriction site was designed upstream of *MeGLYI-13* gene CDS, and an *EcoRI* restriction site was designed downstream of it. Then, the *MeGLYI-13* gene was inserted into the pYES2 vector by double enzyme digestion. The pYES2–*MeGLYI-13* vector was then transferred into the INVSc1 yeast strain via the PEG/LiAc transformation method. To investigate the role of *MeGLYI-13* in response to excessive iron stress, the pYES2–*MeGLYI-13* and control pYES2 transgenic yeasts were treated by SC/Ura liquid medium containing 20 mmol/L of FeCl_3_ for 6 h. After treatment, the bacterial solution was diluted in six gradients: 10^−1^, 10^−2^, 10^−3^, 10^−4^, 10^−5^, and 10^−6^. Then, 5 μL of the diluted bacterial solution was taken from each sample, and the sample was spotted on SC-Ura medium (containing 2% galactose). After incubation at 28 °C for two days, the survival differences between the colonies were photographed and compared.

To further study the function of *MeGLYI-13* in resistance to excessive iron stress, the full-length cDNA sequence of the *MeGLYI-13* gene without the termination codon was connected to the plant expression vector pCAMBIA1300-35S-*GFP* by the homologous recombination method. The obtained vector pCAMBIA1300-35S-*MeGLYI-13*:*GFP* was transferred into *Arabidopsis* by the agrobacterium-mediated pollen tube immersion method. Three positive lines (OE1, OE2, and OE5) were selected for subsequent treatment. The seeds of transgenic and wild-type *Arabidopsis* were disinfected and germinated on 1/2 MS solid medium, vernalized at 4 °C for two days, and then kept at 22 °C under 12 h light/12 h dark conditions for one week. The seedlings were then transplanted onto 1/2 MS containing different concentrations of FeCl_3_ (0, 200, 300, or 400 μmol/L) for excessive iron stress treatment, and three biological replicates were set. Twelve days later, the phenotypic changes and root length were measured. To further understand the tolerance of *MeGLYI-13* transgenic *Arabidopsis* to FeCl_3_ stress, WT and *MeGLYI-13* transgenic *Arabidopsis* were transplanted into pots for 20 days and then watered with 2 mmol/L of FeCl_3_ for another 20 days. The changes in phenotype and physiological indexes such as superoxide dismutase (SOD) activity, malondialdehyde (MDA) content, catalase (CAT) activity, glyoxalase I activity, and peroxidase (POD) activity were then measured.

## Data Availability

Not applicable.

## Supplementary Materials

The following supporting information can be downloaded at: https://www.mdpi.com/article/10.3390/ijms23095212/s1.
